# Gender dynamics in the donation field: human tissue donation for research, therapy and feeding

**DOI:** 10.1111/1467-9566.12803

**Published:** 2018-08-13

**Authors:** Julie Kent, Maria Fannin, Sally Dowling

**Affiliations:** ^1^ Department of Health and Social Sciences University of the West of England Bristol UK; ^2^ School of Geographical Sciences University of Bristol Bristol UK; ^3^ Department of Nursing and Midwifery University of the West of England Bristol UK

**Keywords:** biobanks, blood donation, breastfeeding, abortion, gender, pregnancy, placenta

## Abstract

This paper examines how gender dynamics shape human tissue donation for research and for human health. Drawing on research investigating the donation of different types of bodily tissues including blood, plasma, breastmilk, cord blood, foetal tissue and placentae we consider how and why women and men are viewed as different kinds of donors. We situate these donation practices within a broader understanding of gender difference to explain why any sociology of donation needs to take account of gender. In so doing we explore how tissue derived from the bodies of women acquires value in distinctive ways and for distinctive purposes and reasons. Within these gendered bioeconomies of donation, the supply and demand for tissue is structured by social understandings of maternity, parental responsibility, and risk, which in turn affect the experiences of donors.

## Introduction

The donation of human tissue is often associated with notions of altruism, with giving without expectation of immediate return, and with care. For example, since early discussions of blood donation and the ‘gift relationship’ (Titmuss 1970), blood services reliant on voluntary unpaid donation have been understood as building social solidarity and as tied to citizenship and community belonging. The presumption that donation builds social solidarity and expresses the ‘care’ of members of a society towards each other shapes donation practice in multiple ways, from appeals by national donation systems to increase donor participation rates to the handling and processing of donated materials. Yet the gendered aspects of donation often remain under‐acknowledged even in critical literatures on donation.

In this article our focus is on how examining donation through the analytical lens of gender is important for understanding the ethical valuations and notions of risk associated with donation and with donated materials. Gender is also an important analytical framework for understanding moral hierarchies in which some donors and donations are viewed as ‘good’ and therefore desirable but others are viewed as less desirable. Despite general appeals to the ‘gift’ and to universal notions of altruism in which donation is open to all and gifts are equally valued, we argue that donation is a deeply gendered practice. To demonstrate this, we examine how gendered expectations and norms surrounding the donation of aborted foetal tissue, placental tissue, umbilical cord blood, breastmilk and blood shape the value of these donated tissues (we use this term to include cellular materials, organs, fluids, and gametes). By ‘value’ we refer not only to how donated materials may acquire ‘exchange value’ within human tissue markets (where they exist) but also how they have ‘biovalue,’ which refers to the ‘surplus value of vitality and instrumental knowledge that can be placed at the disposal of the human subject’ (Waldby 2000, 19, see also Waldby and Mitchell 2006). In this sense, we understand value as the multiple social, cultural and ethical assessments attached to human tissues. This framework of value as more than market‐ or exchange‐based, permits us to examine how different presumptions of ‘risk’ may be attached to donated materials. A sensitivity to the multiple meanings of value also allows us to assess how ethical valuations of tissues as ‘just waste’ or as ‘precious resource’ shape how donors are understood to be attached to, or detached from, their bodily materials. We suggest that a sociology of donation requires exploring, as we do in this essay, how the subjects, relations and practices of donation are gendered. It also requires developing analytical tools for understanding gender dynamics in the donation field.

As our discussion below highlights, the gender dynamics of donation shape expectations regarding the interest of donors in their donated material. These dynamics may differ depending on the uses of such materials for distinct purposes: research, therapy or feeding. Human tissue collection and use for research operates within a legal and ethical framework that needs to be understood as distinct from, but connected to, tissue collection for transplantation or transfusion. In the EU, regulations governing the therapeutic use of donated tissue exclude the use of tissue for research (Kent and ter Meulen 2011). However, donors in some circumstances may be encouraged to consider their donation as beneficial for both research and for therapy (Busby 2010). Biological materials provided during clinical care may, in some contexts, be transformed into research resources (Pulley *et al*. 2008). Research on gamete donation demonstrates how tissues such as sperm and eggs collected initially for fertility therapy may later be donated for research (Waldby and Mitchell 2006). These examples make clear that the trajectories of donated tissues are complex. The gender dynamics of donation may differ depending on the use of donated tissues or may change as tissues are transferred from one use to another. Indeed, the tissues we discuss in this article are used for more than one purpose: peripheral blood and umbilical cord blood are used therapeutically in transfusion but also in research (Newcomb *et al*. 2007). Foetal and placental tissue are used in both research and for therapeutic transplantation: foetal tissue is used to treat diabetes and Parkinson's disease (Barker *et al*. 2013, Fine 1994) while placental tissue is an established source of grafts for treating ophthalmologic disorders (Silini *et al*. 2015). Placental tissue is also the focus of current experimental treatments for a range of skin conditions (Nevala‐Plagemann *et al*. 2015). Finally, breastmilk is primarily used for infant feeding but is also collected for research (often using unused milk donated to breastmilk banks), for example in studies of environmental toxin exposure (Hooper and She 2003) and of breast cancer (Shenker 2015, see also the Breastmilk Epigenetic Cohort Study (BECS), and UMass Breastmilk Lab (http://www.breastmilkresearch.org/donate-breastmilk-for-research/). Blood is donated by both men and women; the other tissues are donated by women.

While recognising that there are multiple ways in which these donated materials may be used, our discussion focuses on how gender shapes the initial dynamics of donation of a range of tissues for specific uses. In the field of therapeutic applications, drawing on our research we consider how gendered notions of ‘risk’ construct women as blood donors and are features of the cord blood economy. We discuss the research use of aborted foetal tissue in stem cell science and of placental tissue in a research biobank, highlighting how gendered understandings of maternity shape the social and ethical value attributed to these tissues. Finally, we consider breastmilk donation for infant feeding (we do not discuss here other exchanges of breast milk for adults seeking nutritional supplements or for other reasons) and how maternal donation of breast milk is only visible when breast milk moves between unrelated mothers and infants. As we shall see when a birth mother feeds her own child this is not seen as ‘donation’ even though milk flows from her body to the infant. This work therefore provides an opportunity for analysis of how gender is performed in each of these sites. We discuss extant literatures on donation of these tissues, as well as our own empirical research, to support our argument that donation is a gendered practice. To structure our discussion, we address three specific questions in relation to the gender dynamics of donation:

- Why is gender important in developing a sociology of donation?
- How might comparisons of findings from our research on different types of bodily tissue illuminate gender dynamics in this area?
- Why is it important to think about concepts of risk, safety and benefit, in relation to human tissue donation, as gendered?

## A gendered sociology of donation

Gender as an analytical concept is often used to elucidate how societies make distinctions between the roles, social expectations, behaviours, and bodily performances of men and women. For us it is important to think about how gender constructs both the experience of donation, the valuation of donated materials and the relations between donors and recipients. Extending the emphasis on altruism and care as key presumptions made regarding donors and donation, we might suggest that if women are assumed stereotypically to be more ‘caring’ than men, then could it also be assumed that women in general are more likely to donate? Researchers seeking to answer this question often compare blood or organ donation rates for men and women. For example, a study of blood donation in the US noted that in the 1970s and 1980s, more men donated blood than women. However, during the 1990s the proportion of women donating blood began to increase (Gillespie and Hillyer 2002). In the UK, ‘in 2013, a significantly higher proportion of blood donors were females (54.1% vs. 45.9%; p < 0.001)’ (PHE 2014). In a study of attitudes towards organ donation in the UK, we see that ‘women aged 35‐54 years are significantly more committed to donating their organs than any other demographic. Two‐thirds say they are definitely willing to donate all of, or some of, their organs’ (Optimisa Research 2013). For living kidney donation, more donors are women (53%), while the majority of recipients are men (57%) (NHSBT 2016). Academic literature comparing donation rates of men and women suggests that in general, and across comparative country contexts, more women donate organs than men, and more men are recipients of donated organs than women (Puoti *et al*. 2016, Steinman 2006). Data from the UK National Health Service on cadaver organ donation and transplant in 2015–2016 indicates that 51% of organ donors after brain death and 61% of organ donors after circulatory death are men. Cadaver organ donation is of course influenced by the decisions of relatives of the deceased, although the nature of how gender dynamics figure into the relationship of the deceased to family decision‐making is still underexplored in the scholarly literature (Martinez *et al*. 2001).

The data on demographic characteristics of donation reveal disparities between the proportion of women and men donating specific tissues. These findings regarding organ and blood donation for therapeutic use suggests that gender differences matter but whether, and how they matter is uncertain. It is also unclear whether the gender dynamics of organ donation are relevant to donation of other tissues, or for non‐therapeutic uses. What is needed in order to analyse this descriptive data is research on how donors view donation, and on the extent to which social and cultural dynamics – such as gender – inform their views on, and participation in, donation (Lipworth *et al*. 2011).

In her 2009 article Rene Almeling asks, ‘Does the social process of assigning value to the human body vary based on the sex and gender of the body being commodified?’ (2009: 39). Almeling addresses this question by comparing egg and sperm markets in the US and concludes that ‘the differential expectations of and compensation for egg and sperm donors are generated by gendered assumptions about women and men, including their differential “investment” in reproduction’ (2009: 45). In her study, women egg donors recruited to donation programmes were expected to be altruistic, while sperm donors were expected to be donating for money. Almeling's research is illuminating because she highlights how the sale of human eggs and sperm, and their respective values as commodities in US gamete markets, draws on gender stereotypes and asymmetries between two types of transactions, despite both being part of a commercial market. Her comparative approach enables her to think through how, in the context of the commercial market for gametes, different business models and pricing structures developed for these exchanges. In the recruitment of egg donors, having the ‘right motivation’ to donate means demonstrating altruistic motivations – the narratives of ‘giving and helping’ others were emphasised in recruitment drives. In contrast, strategies for recruiting and marketing by sperm banks emphasised financial incentives as men were thought to be motivated by money to donate sperm (Almeling 2011). Building on existing gender analysis of gamete donation (see Franklin 2013, Spallone 1989, Strathern 1991), Almeling's analysis highlights how both the economic and cultural valuations of sperm and eggs are intertwined and are structured by gender differences.

Other studies of gamete donation, especially egg donation, have highlighted how gender and other social and economic inequalities shape patterns of donation. For example, the cross‐border travel of eggs to wealthier women from those from poorer backgrounds (Nahman 2013) suggests that financial incentives can shape donation experiences for some groups of women. Indeed, it has been argued that women should have property rights to their ova and entitlement to payments, even though in some contexts ethical frameworks prohibit this (Dickenson 2007). Meanwhile, other donation schemes structure payment as ‘compensation’ in an effort to foreground narratives of ‘giving and helping’ rather than ‘selling’ eggs (Haimes *et al*. 2012, Waldby *et al*. 2013). ‘Egg sharing’ draws on a narrative of gifting to, and caring for, others – enough to ‘share’ one's eggs. This framing has been drawn on to recruit egg donors to stem cell research programmes by eliding ‘donation’ for treatment with ‘donation’ for research (Roberts and Throsby 2008). Indeed, any sociology of donation needs to take account of such distinctions for, as we will discuss, donating tissues for research (or to a biobank) constitutes different relationships than donating for therapy or feeding.

In recent discussions about donating blood for research purposes, those who are already blood donors for therapeutic use indicate they may be less willing to also donate blood for research (Cohn 2016). Motivations to become blood donors (commonly to ‘help others’) thus do not so straightforwardly transfer to donating blood for research purposes. However, Gill Haddow's (2009) research on the creation of a Scottish population biobank affirmed that gendered understandings of women as ‘kin‐keepers’ shaped the recruitment of donors to the biobank. Women were likely to recruit other family members to become donors, affirming gender norms that ascribe to women more responsibility for the care of members of their family. These examples suggest that although gender dynamics are critical for understanding the differences between men and women's donation of materials such as gametes, organs and blood, a gender‐sensitive analysis is also necessary for understanding how different ethical valuations shape the donation of ‘women's tissues’ such as placenta, foetal tissue and breastmilk. In this sense, donation is a social practice informed by presumptions about the body, kinship, community, and exchange that are inextricable from how gendered notions of maternity, femininity, and relations with others – as well as gender difference – are made. As we will discuss below, the donation of foetal tissue, placenta and umbilical cord blood makes women donors’ connections to an ‘other’ important. The relational status of these tissues means that women's connections to others are considered matters of regulatory, social and economic significance in ways that are both different from donations of other tissues as well as donations from men's bodies. We highlight however that these differences are not simply ‘biological’ but profoundly shaped by gendered expectations surrounding motherhood and maternal responsibility.

## Gender dynamics of blood, cord blood, placenta, foetal tissue and breastmilk donation

We focus in the following sections of the paper on five different tissues: peripheral blood, umbilical cord blood, placental tissue, foetal tissue, and breastmilk, to identify how gender operates at both the point of procurement and use. We synthesise research in these areas thus enriching sociological understanding of donation across diverse sites, between different social groups and for different types of use.

### Blood and cord blood donation for transfusion and transplantation

Our previous research investigating blood economies highlights how gender relations structure recruitment of both blood and plasma donors. For many blood services the preferred blood donor is young, white, heterosexual and male. In some countries women are actively discouraged as blood donors or excluded from blood donation (Chattopadhyay 2006, Mumtaz *et al*. 2012). Despite commonly held beliefs that voluntary, unremunerated national blood services were founded on a social contract that binds communities together through appeals to universality and social citizenship, blood donation is a highly politicised issue. Within transfusion science we found that ‘gendered bodies are both naturalised and made invisible in a discourse which relies heavily on universalising myths that seek to value blood and plasma donation as a social and public good, and ties donation to citizenship and solidarity’ purporting that all donations are valued equally (Kent and Farrell 2015). Rather, through the practices of categorising, sorting, screening, testing and matching, transfusion science (like transplantation science) produces difference. In so doing biocultural categories of racial and ethnic difference (Kierans and Cooper 2011) and gender difference (Kent and Farrell 2015) are produced. These categorisations and associated risk management strategies (discussed below) in turn construct ‘moral hierarchies’ which exclude some social groups, that is some bodies are seen as ‘good’ sources of donated material and these donors as virtuous, while others are ‘undesirable’ and less virtuous (for example because they engage in same‐sex relationships).

In a previous study, it was suggested that the experience of blood donation was also gendered, that for male donors it was ‘just a product’ while for women blood donation formed a relationship with the recipient (Waldby *et al*. 2004). However, in the plasma products industry, male plasma has historically been more highly sought after and valued because it is perceived as a less ‘risky’ product than plasma from women. Two examples demonstrate how gender operates as an organising principle in blood and plasma processing. First, in the UK since 2003, a policy of ‘male donor preference’ has been adopted meaning that all women's plasma obtained through blood donation is usually discarded (i.e. unless there is a shortage of male plasma) because it is associated with a potentially higher incidence of Transfusion Related Acute Lung Injury (TRALI) attributed to high levels of antibodies following pregnancy. The reproductive potential of women's bodies impacts on how they are understood as blood and plasma donors: women are identified as potentially being or having been pregnant at the point of donation and thus at risk of adversely affecting the recipients of their donated plasma. Women are thus constructed as more risky bodies and the maternal body as representing a threat to blood and plasma product safety (Kent and Farrell 2015).

Second, in the prevention of Haemolytic Disease of the Foetus and Newborn (HDFN), a plasma product, Anti‐D, derived from male plasma is administered to pregnant women who are blood group Rh negative regardless of whether they need it, exposing them unnecessarily to the potential risks of contamination that accompany receiving any blood product. Women are given anti‐D when they do not need it because there are institutional priorities saying it is too difficult to distinguish between women who need it and women who do not. Women are put at risk because it is more ‘efficient’ to give this blood product to all women, regardless of whether they need it (which could be determined by foetal genotyping) (Kent and Farrell 2015, Kent *et al*. 2014). Gendered notions of mothers’ responsibilities to broader population health imperatives thus configure their bodies as more exposed to risk.

We argue that this distinction made between men and women's plasma, the presumptions that women's donations carry more risk because of their potential to be or to have been pregnant, and exposure of pregnant women to greater risk, means that ‘plasma technologies and products are inscribed with gender’ (Kent and Farrell 2015). These examples highlight how men and women are regarded as different kinds of donors, and indeed recipients, of biological materials. They also highlight how different valuations of blood and plasma are derived from these differences: women's ability to become pregnant, regardless of their sexual or health histories, shapes the perceived therapeutic value of their tissues and situates their bodies as more risk‐laden than men's, while being pregnant exposes them to risks that go under‐acknowledged (and indeed could be eliminated, see Kent *et al*. 2014). These gender differences, in which bodies are presumed to be more, or less risky, are produced through practices within transfusion science and the plasma products industry. Indeed ‘gendered bodies are produced through the discursive and material practices within national blood services’ (Kent and Farrell 2015). What these examples also highlight is that crucially ‘transfusion science draws on a set of rationalities about the immune system which have emerged within a specifically gendered historical context’ (Kent and Farrell 2015). Theories about the ‘connections’ between the maternal and foetal body and the consequence of these underpin understandings of both blood and plasma donation and transfusion. They also shape the value attached to materials from different bodies. In these examples, male blood and plasma is construed as having higher therapeutic as well as market value and is made more readily available for commodification.

Donation of umbilical cord blood affords another site for exploring how donor relations are gendered. Cord blood is construed as the mother's tissue within the UK regulatory context and can be donated for use in the treatment of blood disorders, such as leukaemia, sickle cell disease and thalassaemia. Since the 1990s, it has become widely used in place of bone marrow transplants. It is estimated that since 1998, 20% of stem cell transplants carried out to treat blood disorders have been from umbilical cord blood, and in Japan, the proportion is closer to 50% (Gluckman 2009). In the UK, public cord blood banks were established in areas where a high proportion of the population of pregnant women were from Black and Minority Ethnic (BME) groups since it is these groups for whom a bone marrow match was more likely to be difficult to obtain. Or rather, the claim that there is unmet need amongst BME groups took on the status of fact in the shaping of governance arrangements for the sector (Williams 2015). As part of a strategy to improve the availability of cord blood for transplantation, these groups have been targeted for recruitment.

However, it has been suggested that the expansion of cord blood banking services and awareness of the therapeutic uses of cord blood mean that other women who are not targeted for public cord blood bank donation articulate what Machin *et al*. (2012) call a ‘right to donate.’ Women's expectations about cord blood donation appeared to outstrip the infrastructure for collection by public banks. Some women, believing that they should have a right to donate to the public bank, not least because it might benefit their family at a future date, were critical of the under‐resourcing of public cord blood banks. This rights‐based approach to donation was understood in terms of the benefits a donor's family might receive and as discriminatory if collection and storage wasn't possible for all who wanted it. In this example, the ‘rights’ of the women donors were constructed in terms of ‘the potential future health needs of donors, their children and their family’ (Machin *et al*. 2012,: 301) and draw on gendered understandings of mothers’ responsibilities to act as gatekeepers for their family's health and to make ‘wise choices’ on behalf of their children (Fannin 2011, 2013).

The privatisation of cord blood banking arguably contributes to women's attitudes in the study described above. We suggested previously that commercial or ‘private’ cord banks are promoting a kind of ‘hoarding’ of biological materials by encouraging parents to withdraw their donation from public circulation. Instead, an individualised form of saving for the future ‐ protecting one's family from potential unknown risks as a kind of insurance ‐ is the basis of ‘donation’ and storage (Fannin 2013). In this way, banking is viewed as a way for mothers to invest in the future and in so doing act as responsible parents in ways that cohere with other gendered expectations surrounding motherhood. Interestingly in this analysis, responsibility rather than rights is the basis of actions. As Haw (2016) suggests, an ideology of ‘intensive mothering’, in which mothers devote considerable financial resources and time to their child's intellectual, social and physical development, is reinforced by private cord blood banks and women do a great deal of work to donate (see also Hays 1996). Women donors must conform to regulatory requirements in the initial collection of cord blood, and Haw notes that this burden of work is often underestimated by women themselves and not acknowledged by cord blood companies. Gendered assumptions about women's willingness to assume responsibility for their child's present and future health make this burden invisible. This kind of donation ‘[expands] the temporal frame of health concerns mothers are responsible for managing (i.e. the future health of their unborn child) and the way in which these health conditions are managed (i.e. by having a biological treatment if and when it is needed)’ (Haw 2016: 47‐48). Donation is also reconfigured as a process of proxy generational giving and receiving. The mother/parents decide(s) to store or retain the cord blood on behalf of their child and family. In commercial banking, these relational aspects of donation are central but are restricted to personal and kinship ties in contrast to the wider community. Claims for a universal cord blood collection system thus mobilise both public health benefits and private interests. Women seek to donate cord blood in the expectation that the state will provide a ‘match’ for their families, combining expectations of the state's responsibility and rights, altruism and personal benefit (Machin *et al*. 2012).

This discussion highlights the gendered aspects of donation practices surrounding peripheral blood and cord blood stem cells. In all of these sites, a woman's status as mother, or as potential mother, shapes donation experiences and expectations about the ‘right to donate’ and about the assumption of responsibilities to donate or store tissues. Reproduction (rather than production) defines these relationships, and gendered notions of donors as (potential) mothers and of maternal responsibility profoundly shape the process of donation. As in Almeling's discussion above, it is women's connections to others that crucially influence how donation is understood, whereas for men, donation is also about their market provision and participation.

### Placenta and aborted foetal tissue in bioscience research and biobanking

Placental tissue and aborted foetal tissue are ‘products of conception’: tissues produced specifically from pregnancy. Products of conception are like ova in the sense that they are considered part of women's bodies, but at the same time unlike ova because they are not genetically identical to the mother (and indeed can be genetically male). Aborted foetal tissue has been collected and used in stem cell science, for experimental transplantations to treat degenerative brain disease and for the study of embryology, for many years. Foetal tissue travels from the abortion clinic to the stem cell laboratory and is transformed from ‘waste’ or a ‘corpse’ into a bio‐resource generating new types of bio‐objects (Kent 2008). Similarly, placental tissue collected at birth is usually viewed as ‘waste’ that can be transformed into a research tool and scientific object.

While embryo donors who undergo IVF treatment are constructed as helping others (other women or scientists), that is, as being donors motivated more by altruism, women donors of foetal tissue were seen as ‘different types of donor’ from donors of embryos. In our research on foetal tissue donation, women donating foetal tissue were more likely to be represented as ‘unthinking’ with no long‐term commitment to research or abortion care (i.e. medical science and practice). Women seeking pregnancy termination often did so in difficult circumstances and were thought to ‘just want to get the abortion over.’ Consent procedures were based on assumptions that women terminating pregnancies should not be given specific information about the research use of foetal tissue in case it influenced their decision to have an abortion, and that when asking for consent the women were unlikely to want to know details of what might happen to the foetus as it would be too distressing for them. For women themselves it was important that the foetus no longer continued to live after the termination. While it has been a legal requirement in the UK to separate the decision to have an abortion from the decision about whether to donate the tissue, the method of abortion could be modified to suit the needs of the research if the woman consented (Kent 2008, Pfeffer 2008).

Analysis of how women's reproductive labour is tied to notions of wastefulness (Ariss 2003) leads to a view that ‘women who terminate a pregnancy are especially “wasteful” and by inference morally suspect’ (Kent 2008). Constructed as waste tissue, the dead foetus becomes available for re‐use and latent bio‐value is released. And, by donating foetal tissue for research, women thought some good could come from the difficult experience of abortion. Tissue donation offers the potential to ‘do good’, to redeem oneself. It could also assist with grieving. The ambiguous legal and moral status of a foetus contributed to a situation where at times it was the woman who was regarded as the ‘donor’, while at others the foetus itself was viewed as an organ donor. A woman's decision to donate an aborted foetus was both likened to donating an organ and as very different, because she first had to decide whether to end her pregnancy. The ethical sensitivities surrounding both pregnancy termination and donation of an aborted foetus for research use were intertwined. Indeed, moral valuations of women who have abortion translated to valuations of donated foetal tissue as more likely to be ‘contaminated’ (Kent 2008).

Comparison and contrast between aborted foetal tissue donation and placental tissue donation reveals how gender dynamics shape donation of these tissues that are both derived from pregnancy. Like the ambiguities surrounding the identity of the ‘donor’ of foetal tissue (mother or foetus), the nature of the placenta's formation during pregnancy creates ambiguities in the regulatory, medical and donor conceptions of it. The placenta is commonly presented in medical literature as genotypically foetal tissue, however the placenta is composed of both maternal and foetal tissue: although the umbilical cord and membranes are foetal, the placental bed includes maternal and foetal tissues in close proximity. In the UK, the Human Tissue Act (2004) includes the placenta as a ‘relevant material’ and describes the placenta and its membranes as one of the ‘non‐foetal products of conception’ along with amniotic fluid and the umbilical cord. For the purposes of the Act, placental tissue is considered the mother's tissue. The Human Tissue Authority makes a distinction, however, between the procedures that should be put in place for the disposal of term placentas and the procedures that hospitals should carry out for the disposal of the remains of pregnancy, including placental tissue, that may be the products of pregnancy loss or termination (Human Tissue Authority 2015). Given the perceived accessibility of term placental material – placentas collected at full‐term birth – few studies have been carried out on perceptions of the demand or supply of this material for research. The donation of the placenta is presented in some contexts as free of the ethical controversies that surround the donation of other materials derived from pregnancy, such as embryonic or foetal material, and thus term placental material is valued in part for the perceived lack of ethical controversy surrounding its donation and use. Like umbilical cord blood, donation of placentas to biobanks for storage for future personal use is also advertised by commercial banking companies in the US (see https://www.lifebankusa.com).

Very few studies have been carried out with donors of placental material or scientists regarding their perceptions of the placenta (Fannin and Kent 2015, Yoshizawa 2013). A study of one UK biobank established over twenty years ago found that the placenta collection was viewed as ‘a unique archive of the connection between the mother and the child during pregnancy’, as a kind of ‘connective tissue’, ‘a distinctive kind of bio‐banked material precisely because it is more than foetal material’ (Fannin and Kent 2015). Its scientific value related to what it could tell researchers about a child's future health and the ‘foetal environment’ *in utero*. For women donors, its meaning related to the experience of childbirth and it was understood as a biographical object. The birth rather than the placenta was significant for them and women were described as more interested in the baby than what happened to the placenta. Ambivalence about the ownership of the placenta, as belonging to the mother, child or shared, meant that permission to ‘retain’ the placenta rather than to ‘donate’ the placenta was sought when they were first collected in the early 1990s.

Survey and interview research with postpartum women in a Brazilian maternity hospital suggests that women are supportive of requests for their consent to donate placental material for research use, but would like more information about how the placenta might be used and what happens to it after birth: ‘Disinterest in seeing the placenta at birth, and a lack of spiritual valuation, does not mean women are indifferent to the organ or its final fate. These women do not actively participate in the discarding of the placenta, despite what scientists and clinicians may claim’ (Yoshizawa *et al*. 2015: 81). In another study, 25 women were interviewed about their views regarding placental donation for a placental perfusion study; the authors found that most women viewed the placenta as ‘waste’ and ‘felt there were no unethical aspects at all’ to the donation of placental material for research (Halkoaho *et al*. 2010: 689). The few studies carried out suggest that women donating placental material consider the placenta to have already ‘done its job’ during pregnancy (Fannin and Kent 2015).

Donors to biobanks are increasingly the targets of messages surrounding the ‘wise use’ of materials that would otherwise ‘go to waste’ (Tupasela 2011), and increasingly placental tissue has been attributed speculative value in these terms. Analysis of the discursive framing of biological materials derived from pregnancy as in need of ‘efficient’ use suggests that the gendered construction of the responsible mother now includes her role as a wise saver of potentially valuable biological materials for her family's benefit. Comparing foetal tissue and term placental tissue donation reveals some similarities, and striking differences, in how these materials from pregnancy are valued. First, despite foetal tissue and placental tissue both being the products of conception, the circumstances of their collection are distinct: for foetal tissue donation, tissues are collected at the termination of a pregnancy; for term placental tissue donation, tissues are collected at the culmination of a pregnancy. In both cases the perceived wastefulness of reproductive tissues is the basis for efficiency gains secured via donation. However, ‘socially responsible’ mothers donating placental tissue for the potential benefit of (their) child's health occupy different social positions and relations from those of women donating foetal tissue for stem cell science after an abortion. In donating placenta, mothers express the connection with their child and with a contribution deemed valuable because it represents this connection. In the case of placental biobanks, this contribution also constitutes their participation in a community of donors (Fannin and Kent 2015). Whereas for women who have elected to abort their pregnancy – separation from the foetus is key and despite the high numbers of women undergoing abortion, their social position can be characterised by marginalisation. In these circumstances donation construed as a means of recompense is very different. Underpinning both experiences, however, are highly gendered meanings and valuations of motherhood, pregnancy, childbirth and abortion in which term placental material is deemed to originate from a ‘desired’ pregnancy while aborted foetal tissue is from an ‘undesirable’ pregnancy. In this case, understanding how pregnancy is laden with moral and social values surrounding motherhood is critical to understanding how the donation of foetal and placental tissue is shaped by gender dynamics.

### Breastmilk for infant feeding

The fifth site where we explore donation is human breastmilk for infant feeding. Our aim is to contrast donation for feeding with research and transplantation use. Human breastmilk is produced from biologically female bodies to feed human babies; men also have mammary tissue and milk ducts and some, through stimulation, have produced milk although this is rare and of unknown nutritional benefit. Breastmilk is also sold online and used for purposes other than feeding babies (Steele *et al*. 2015). Our focus here, however, is on the use of breastmilk for nutritional purposes and on the donation of breastmilk through milk banking and milk sharing.

In many countries, since the early 20^th^ century, formal ‘milk banks’ have collected and distributed donor milk to babies who were premature or whose own mothers could not feed them or were unwell. Many of these banks, worldwide, closed in the early to mid‐1980s in relation to fears around contamination with HIV and other blood‐borne viruses (Swanson 2014). In the UK, a resurgence of milk banking has taken place, supported by the UK Association for Milk Banking (UKAMB 2014). Clinical Guidelines from the National Institute of Health and Care Excellence (NICE 2010) support the operation of donor milk banks, providing guidance on how milk is tested, transported, treated, stored, labelled, and tracked; who should and should not donate milk; how donors should be screened and who should receive donated milk. Much of the history of milk banking demonstrates the importance of the ‘mother‐to‐mother’ aspects of donation; the appeal to women's generosity and references to ‘the milk of human kindness’ emphasise the relational aspects of donation. However, women's milk has also been seen in other ways; Akre *et al*. (2011) note that there has always been suspicion around women's milk from early concerns about wet nurses (morals, lifestyle, diet) to present day fears about disease transmission (concerns which are magnified by using the Internet to facilitate exchange). Swanson (2014) notes several ways in which the early development of milk banking (particularly in the US) drew on specific constructs of femininity, a ‘gendered vision of caring’ and a language of ‘intimate relationships and uncompensated caring’ (2014: 168). Donating breastmilk is perceived as a ‘maternal gift’; attempts in the US in the 1980s to charge for breastmilk were interpreted by donating women as ‘commodifying motherhood’ (2014: 182).

Historically, women have informally ‘cross‐nursed’ (Shaw 2007) the babies of other women, often relatives or friends, or have sold their milk through ‘wet nursing’ arrangements. Despite the resurgence of milk banking in many countries women in the twenty‐first century sometimes seek breastmilk for infants who are not eligible for banked milk. They may refer to the WHO guidance which suggests that best alternatives to a mother's own milk are milk from a wet nurse, or from a human‐milk bank, with the last option ‘a breastmilk substitute [formula milk] fed with a cup’ (WHO 2003:10).

‘Milk sharing’ – the ‘commerce free practice in which a donor gives expressed breast milk directly to a recipient family for infant feeding or breastfeeds a recipient infant’ (Palmquist and Doehler 2015: 1) has rapidly increased in recent years as the use of the Internet and social media enables women to make ‘milky matches’ (Cassidy 2012). It is variously known as peer‐to‐peer milk sharing, ‘informal’ milk sharing and private arrangement milk sharing (PAMS); the language of ‘sharing’ seems to be used as much as that of ‘donation’. Milk sharing via the Internet has moved ‘from private practice to public pursuit’ (Akre *et al*. 2011: 1), with breastmilk a ‘mobile biosubstance…able to travel on its own apart from the lactating body’ (Boyer 2010: 5) in novel ways. In the past, a mother had to know another who was lactating, and who was prepared to donate to or feed their baby; now via the Internet women can connect with each other to donate or receive milk. Although Internet peer‐to‐peer sharing is often portrayed as anonymous, the research to date contradicts this; most donors seek information about recipients and vice versa, form relationships and know about the families and babies they are donating to, influencing their motivation (Gribble 2013, Palmquist and Doehler 2015).

Milk sharing in this way has been the subject of little research outside the US (Palmquist and Doehler 2015) but some public health bodies have strongly advised against it (in the US, Canada and France), focusing on ideas of ‘danger’ and ‘risk’. Although not from a regulatory body, these were repeated in the UK context in a *British Medical Journal* editorial (Steele *et al*. 2015). Breastmilk is seen as risky per se ‐ milk donated to milk banks is seen as a biohazard; as noted, women donating milk are subject to a range of tests and restrictions, all suggesting risks to be mediated. Milk banks sometimes use language implying that a mother's milk might even be a risk to her own child by suggesting that milk which has not been processed by the milk bank is not safe (Palmquist 2015). Informal milk donation is particularly controversial as it highlights the dichotomy between ‘breastmilk‐as‐medicine’ and ‘breastfeeding‐as‐pollutant’ (Palmquist 2015: 30).

A small body of research has attempted to quantify the potential risks of donation (Keim *et al*. 2013), although not always distinguishing between milk that is sold and milk that is donated. Recent research has looked at risk in commerce‐free, milk sharing scenarios (Perrin *et al*. forthcoming). Potential risks include disease transmission, microbial contamination (related to poor storage and handling) and those from medication or substance use. Some assumptions in the assessments of risk have been challenged (Geraghty *et al*. 2011, Gribble and Hausman 2012), with on‐going research in the US assessing how women think about, make sense of and mediate the risks in the absence of evidence‐based healthcare guidance (Palmquist and Doehler 2014). Internet sites facilitating milk sharing offer advice on informed choice, donor screening, safe handling and home pasteurisation (see http://www.eatsonfeets.org). These ‘Four Pillars of Safe Breastmilk Sharing’ are drawn on in the American Academy of Nursing on Policy's (2016) ‘Position statement’ for health professionals. No similar advice has been offered to mothers or health professionals in the UK.

There are several ways of viewing the rise in peer‐to‐peer milk sharing, its connection with formalised milk banking and its purpose here as an illustration of a gendered form of donation. Milk sharing is a specifically gendered activity; in the same way that in the early to mid‐twentieth century women rallied together to provide breastmilk to needy babies (Swanson 2014), contemporary women can be seen to be working together to tackle an issue: ‘well‐informed and highly motivated women have begun extending control over the availability and use of human milk’ (Akre *et al*. 2011: 1).

Societal expectations that the exchange of this fluid is hidden and leakages discreetly managed reinforce its status as ‘matter out of place’ (Dowling and Pontin 2017, Dowling *et al*. 2012) and relate to other societal meanings associated with women's bodily fluids. Breastmilk is expected to pass discreetly from mother to baby and is disturbing when it does not (Zizzo 2011). The history of milk banking demonstrates a gradual medicalisation and dehumanisation of an activity shared by women (Swanson 2014); the rise in milk sharing using the Internet can be interpreted as women challenging the medical establishment. Their actions cannot be regulated in any meaningful way, and so mothers have control (Akre *et al*. 2011), with echoes of the long history of mother‐to‐mother support in relation to breastfeeding as illustrated by the organisation La Leche League (Bobel 2001).

Milk banks depersonalise breastmilk, making it more acceptable and removing its negative cultural associations. The process leads from a woman expressing milk from her breasts to the ‘product’ in a milk bank – called PDHM (Pasteurised Donor Human Milk) or BDM (Banked Donor Milk). The process of medicalisation, in which breastmilk becomes a prescribed therapy for neonates, is one in which the women who produce the milk are gradually ‘eradicated’ (Hassan 2010 in Palmquist 2015). Breastmilk separated from its human origins has been shown to be more acceptable to many parents; for some mothers this has the effect of removing the association that it came from another woman: ‘it just makes it…like part of the hospital…You know, not…from somebody’ (Zizzo 2011: 7). However, some women prefer peer‐to‐peer sharing because they dislike the depersonalisation – ‘I hated the idea of my sending my milk somewhere and having it treated like a specimen instead of the life force it is’ (Gribble 2013: 453).

Milk ‘donation’ is usually understood as the transfer of human milk from a lactating woman to someone other than her own infant. Feeding one's own infant is normalised to the extent that this is not construed as a ‘donation’; this is a fundamental part of the act of care associated with mothering. Social expectations that mothers should feed their child if possible underplays the act of ‘donation’ here and appears to gloss over the exchange of bodily fluid taking place. Breastmilk as product and breastfeeding as practice clearly have different meanings (Zizzo 2011). It surely tells us something about the gendering of donation practice that mother's milk for the benefit of one's own child draws on assumptions of self and ‘non‐self’ embodied in the mother/child relationship. Only when made available for wider circulation, via a sharing arrangement or milk bank does the ‘donation’ come into view. Breastfeeding is not clearly delineated from maternity; milk donation in all its forms is seen to be transgressive and controversial, going ‘beyond the confines of biogenetic and gestational motherhood’ (Shaw 2007: 440).

In this example, the commodification of milk as a bioresource seems to loop back to a donation. Unlike cord blood which is separated from the infant, regarded as ‘waste’ and donated by the mother, human breastmilk is not viewed as waste rather it is viewed as belonging to the mother and her baby. Its ‘proper’ use is to feed that baby; moral questions only arise when, and where, it may be distributed outside that relationship. Where those distribution networks are ‘private’, with sharing amongst friendship groups or personalised social contacts this seems to acknowledge the social importance and potential health benefits attached to feeding with human breastmilk. Distribution online and via social media is construed as representing greater potential risks and regulatory challenges. Meanwhile milk banks recruit women donors through techniques that for example suggest that they can ‘give something back’:

“Many of the women who donate milk are giving something back because they were helped themselves but you can donate even if your child has not needed help.” http://www.northwesthmb.org.uk/donate/

Participating in milk bank donation works through notions of ‘giving back,’ reciprocation and gratitude for a gift received. Here the gendered dynamics of sympathy and reciprocity are at work: a child will directly benefit from this gift, not the donor herself. This draws on more than just helping another person (the stranger paradigm of the gift of donation) but on helping another child, or one could say, extending one's maternal ‘gift’ to another woman's child or to another mother and child.

## Conclusion: gendered concepts of maternity, responsibility and risk

In five different sites, we highlight how donation practices are shaped by gendered dynamics of motherhood, maternal responsibility, and risk. Tissues generated by pregnancy – foetal tissue, umbilical cord blood, placenta and breastmilk – are understood as co‐produced, that is produced through relations of both maternal and foetal bodies, that complicate their definition as tissues ‘belonging’ to one person. This in turn challenges presumptions of self and other and shapes understandings of donation. The specific social dynamics shaping the donation and valuation of these materials cannot be ascribed solely to their ‘biological’ difference from materials donated by men. Recognising that certain bodily tissues and fluids such as aborted foetal tissue, placental tissue, umbilical cord blood and breastmilk are produced by pregnant and birthing bodies, we argue that the dynamics of donation and the valuation of these materials are shaped by gendered concepts of maternity/parenting, responsibility and risk. In this way, we bring together insights from donation for different purposes – research, transplantation and feeding – in order to illustrate how gender dynamics shape the value of these donated materials.

Viewing donation as a gendered practice is also important for understanding how concepts of risk, safety and benefit shape the donation field. Medical approaches to women's bodies, particularly in relation to fertility and pregnancy, tend to view women's bodies as particularly risky and their care as informed by calculations of risk (Lupton 1999, Weir 2006). For example, the production of antibodies during pregnancy, as we have seen in the discussion of the gendered donation of peripheral blood, is viewed as a key factor in why women's donations of blood are viewed as riskier than men's. At the same time, some pregnant women receive donated blood products, exposing them to unnecessary risks. The regulatory processes surrounding exposures to risks from donated material are here shaped by the presumption that protection of foetal bodies from potential harm is a risk mothers are willing to take, although as Kent *et al*. (2014) indicate, they are given no viable alternative by their national healthcare service. The regulatory process surrounding women's antenatal care thus reflects broader gendered dynamics in healthcare regulation, where risk is constructed through gender and mediated by institutional policy arrangements.

Gendered understandings of women's bodies as fluid or leaky also inform how donated tissues like foetal tissue, placenta and breastmilk are viewed as ‘matter out of place’ and thus more difficult to contain and regulate. The exchange of breastmilk outside of formal networks and without regulatory scrutiny is viewed as risk‐laden and difficult to control, as it could expose infants to risk from other mothers. Breastfeeding is also an illustrative example of how donation as a concept excludes the feeding of breastmilk by a mother to her own child. Only if the child of another mother receives breastmilk does the concept of ‘donation’ apply. The exclusion of a mother feeding her infant from the concept of donation underscores how donation is imagined on the model of the ‘stranger's gift’ and in doing so, relies on the presumption that maternal‐infant exchanges lie outside the frame of a proper exchange between donor/recipient. The implications of this are that some forms of donation are naturalised as part of mothering practice. The distinction between what is and is not defined as donation suggests that mothers and their infants occupy an ambiguous relation relative to self/other relations of donation.

Our discussion of placenta and foetal tissue demonstrates how dynamics of connectedness and separation shape tissue donation, and how donors’ perceptions of tissue donated from a terminated pregnancy differ from those following a pregnancy brought to term and culminating in a live birth. Foetal tissue and placental tissue are both products of pregnancy, but the views of donors about these tissues are different. These differences can only be appreciated through a gender‐sensitive understanding of pregnancy termination and the construction of maternal subjects in relation to the outcome of pregnancy. The pregnant and breastfeeding body are viewed as a particularly hybrid body more akin to the ‘not‐one‐but‐not‐two’ model of pregnancy articulated by legal theorist Isabel Karpin (1992). Tissues issuing from pregnancy identified as ‘waste’ and viewed as matter out of place may be repurposed through donation but the dynamics of donation will be determined in part by gendered assumptions of the pregnancy from which they originated.

Blood and breastmilk donation illustrate, in different ways, how the relationships said to structure donors’ willingness to donate and the assumptions underlying donation itself are gendered. Blood donation is viewed as integral to the construction of bonds of community within national populations and donating blood is imagined as a civic duty, particularly in times of political conflict or threats to the body politic through natural disaster. Yet donor motivations are informed by gendered presumptions about donation as an act that makes relations or provides a product. The processing of blood products is also informed by gendered presumptions about the different risks associated with women's and men's bodies. Turning to milk donation, in some contexts, milk donation is viewed as creating kinship relations. However, the regulatory processes governing the donation of milk are also shaped by gendered expectations regarding women's bodies as ‘leaky’ and ‘risky’ in ways that recall the gendered dynamics of plasma donation. The framing of donation as a wise use of tissues regarded as medical waste or as unwanted speaks to gendered assumptions surrounding care and of gendered notions of ‘responsibility’ towards others that position women donors in distinctive ways.

Gender also makes a difference to understanding who benefits and how from donation. Sociologists Melinda Cooper and Catherine Waldby (2014) argue that the benefits of the forms of embodied or ‘clinical’ labour carried out by oocyte donors and clinical trial participants are rarely considered beyond the relatively narrow, although not unimportant, consideration of donor/participant consent. The ethical dimension of who benefits from medical research involving human tissues, and how healthcare provision and medical research is embedded in and benefits from uneven global economic development and exploitative labour practices that value gendered forms of bodily labour less than other forms of productive labour, is often elided in considerations of research ethics. Their approach to redefining donor participation in research as a form of labour emphasises how gender difference matters, particularly when calls for donation take place through appeals to ‘women's sense of responsible custody’ (Cooper and Waldby 2014: 115).

Our comparison of donation for different purposes also highlights the importance of developing more nuanced analysis of donation. Donation for research, for therapy or for feeding is informed by different notions of giving and shaped by gendered norms and expectations surrounding citizenship, parental responsibility, and public benefit. Brought together in this paper, our research on diverse tissues donated for different purposes underscores how gender shapes the donation field. Despite general claims in the literature on donation to altruism, sharing, and universal concepts of ‘the gift’, this paper makes clear that such general appeals do not adequately reflect the social realities of donation, in which donating is intimately connected to gendered presumptions and norms. This is as relevant to tissue donated from both men and women's bodies (e.g. peripheral blood), as it is to tissues donated during pregnancy or at birth. A sociology of donation therefore needs to be sensitive to gender difference and to the gendered assumptions that shape the dynamics of donation.
